# Infliximab-induced retrobulbar optic neuritis in a patient with ankylosing spondylitis

**DOI:** 10.3205/oc000232

**Published:** 2023-12-12

**Authors:** Sema Dündar, Mimbay Yaşar, Harun Çakmak, Nefati Kıylıoğlu, Alparslan Ünsal

**Affiliations:** 1 Ophthalmology, Adnan Menderes University, Faculty of Medicine, Aydın, Turkey; 2 Ophthalmology, Bingöl State Hospital, Bingöl, Turkey; 3 Neurology, Adnan Menderes University, Faculty of Medicine, Aydın, Turkey; 4 Radiology, Adnan Menderes University, Faculty of Medicine, Aydın, Turkey

**Keywords:** optic neuritis, infliximab, anti-TNF, demyelinating disease, ankylosing spondylitis

## Abstract

**Objective:**

To present a case with infliximab-induced retrobulbar optic neuritis.

**Case description:**

A 58-year-old woman presented to our clinic with a two-day history of blurred vision in her right eye. She had numerous uveitis attacks previously, and she was on infliximab treatment for ankylosing spondylitis. Her best-corrected visual acuity was counting fingers and 20/25 in the right and left eye, respectively. Optic discs seemed healthy in fundoscopic examination. The right optic nerve showed high signal intensity on magnetic resonance imaging (MRI). Infliximab treatment was discontinued and systemic steroid therapy was started. After the treatment her best-corrected visual acuity improved to 20/20 in her right eye.

**Conclusion:**

Infliximab is a chimeric human-murine monoclonal antibody used in autoimmune diseases. Optic neuritis is a rare but important side effect of infliximab. Thus, infliximab-induced optic neuritis should be kept in mind for patients receiving infliximab treatment.

## Introduction

Tumor necrosis factor alpha (TNF-α) stimulates systemic inflammation and acute phase reaction [1]. Infliximab binds to TNF-α and inhibits its action. Infliximab is widely used for the treatment of inflammatory diseases such as rheumatoid arthritis, inflammatory bowel diseases, psoriasis and ankylosing spondylitis [2], [3]. Infliximab is generally well-tolerated by patients. Most common side effects include infusionrelated reactions like headache, rush and flushing. Demyelinating disorders are rarely seen due to the use of anti-TNF-α agents [4], [5], [6], [7]. Herein, we present a case of infliximab-induced retrobulbar optic neuritis in an ankylosing spondilitis patient.

## Case description

**Figure 1:**
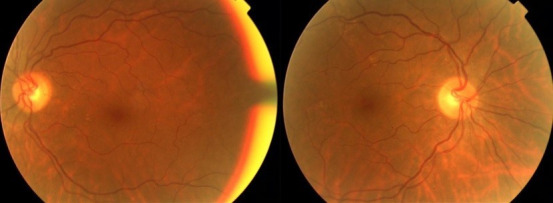
Fundus photograph (FP) of both eyes

**Figure 2:**
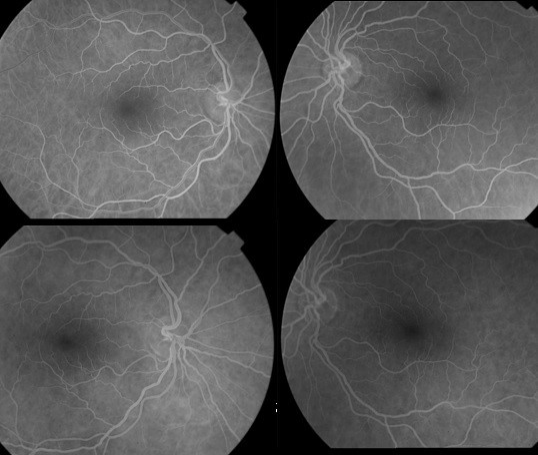
Normal fundus fluorescein angiography findings

**Figure 3:**
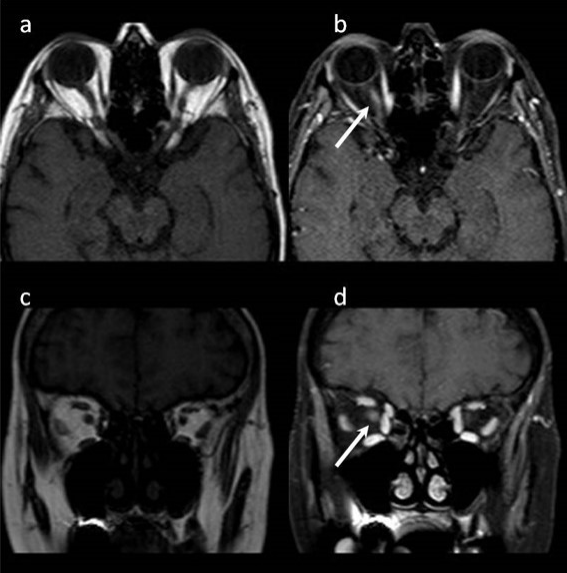
T2 weighted sequence MRI of the brain and orbits revealed high signal intensity in the right optic nerve.

A 58-year-old woman with a 25-year history of ankylosing spondylitis presented to our clinic with a blurred vision in her right eye for two days. Best-corrected visual acuity was counting fingers OD (right eye) and 20/25 OS (left eye). She identified 6 out of 21 Ishihara pseudoisochromatic color plates with the right eye and 21 of 21 with the left eye, and she had a right relative afferent pupillary defect. Her optic discs seemed healthy (Figure 1 (Fig. 1)). Fundus fluorescein angiography and optical coherence tomography findings did not point out any causative pathology (Figure 2 (Fig. 2)). Magnetic resonance imaging (MRI) of the brain and orbits revealed high signal intensity in the right optic nerve (Figure 3 (Fig. 3)). Her neurological examination was unremarkable. Detailed history of the patient revealed that she was receiving infliximab therapy for ankylosing spondylitis. Infliximab treatment was stopped and 1 g/day of intravenous methylprednisolone for five days was started, then switched to oral prednisone 1 mg/kg/day and tapered slowly. Her vision improved gradually. Her visual acuity improved to 20/20.

MRI of the brain and orbits revealed high signal intensity in the right optic nerve (Figure 3 (Fig. 3)). Her neurological examination was unremarkable. The detailed history of the patient revealed that she was receiving infliximab therapy for ankylosing spondylitis. Infliximab treatment was stopped and 1 g/day of intravenous methylprednisolone for five days was started, then switched to oral prednisone 1 mg/kg/day and tapered slowly. Her vision improved gradually. Her visual acuity improved to 20/20.

## Discussion

The patient’s clinical course was consistent with retrobulbar optic neuritis. Alternative diagnoses, such as compressive, infiltrative, and infectious optic neuropathies, were eliminated based on laboratory testing, neuro-imaging and clinical history. The clinical presentation of the patient started after taking three doses of medication. In a case series by ten Tusscher et al., after the third dose of infliximab infusion patients showed signs of anterior optic neuropathy, which was resistant to steroid therapy [8]. Simsek et al. reported a temporary link between the onset of symptoms and duration of drug use [9]. However, it is unclear from published case reports whether the anti-TNF-α agents, cumulative dose, or duration of therapy influences the clinical presentation. This article adds a new case of infliximab-induced optic neuritis to the literature. TNF-α inhibitors are used for rheumatoid arthritis, inflammatory bowel diseases, ankylosing spondylitis and psoriasis [2], [3]. TNF-α seems to have a role in the pathophysiology of demyelinating diseases [10], [11]. Human studies have demonstrated negative effects of TNF-α blocking in multiple sclerosis and precipitating of demyelination has even been described [4]. However, no clear explanation regarding the pathogenic mechanism is available.

Not only infliximab but also other TNF-α antagonists are thought to facilitate autoimmune reactions and demyelinating process [12], [13], [14]. On the other hand, Winthrop et al. evaluated patients in whom anti-TNF-α treatment or non-biologic disease-modifying anti-rheumatic drugs (DMARD) had recently been started [15]. A total of 61,227 patients’ records were analyzed and only six cases of optic neuropathy were noted. Half of the patients with optic neuritis were anti-TNF-α users and the other half were non-biologic DMARD users. They have found similar frequency of optic neuritis with anti-TNF-α agents and non-biologic DMARDs. TNF-α inhibitors are used for inflammatory diseases in gastroenterology, dermatology, neurology, and rheumatology with increasing frequency. In ophthalmology practice, anti-TNF-α agents can be used in ocular inflammation like scleritis and resistant uveitis [16]. Neurological deficits may occur during the treatment with TNF-α inhibitors. Patients who are on anti-TNF-α should be followed on a multidisciplinary basis and properly informed about potential side effects.

## Conclusion

It is important to consider the possibility of optic neuritis in patients who experience sudden-onset visual loss while being treated with infliximab. If a patient using TNF-α blockers experiences visual abnormalities, he/she should present to an ophthalmologist immediately. 

## Notes

### Patient consent

Consent has been obtained from the patient.

### Competing interests

The authors declare that they have no competing interests.
